# Supplementary figures and images for: BnGF14-2c Positively Regulates Flowering via the Vernalization Pathway in Semi-Winter Rapeseed
Source: Plants (Basel). 2022 Sep 3;11(17):2312. doi: 10.3390/plants11172312 (PMC9460199; doi:10.3390/plants11172312)

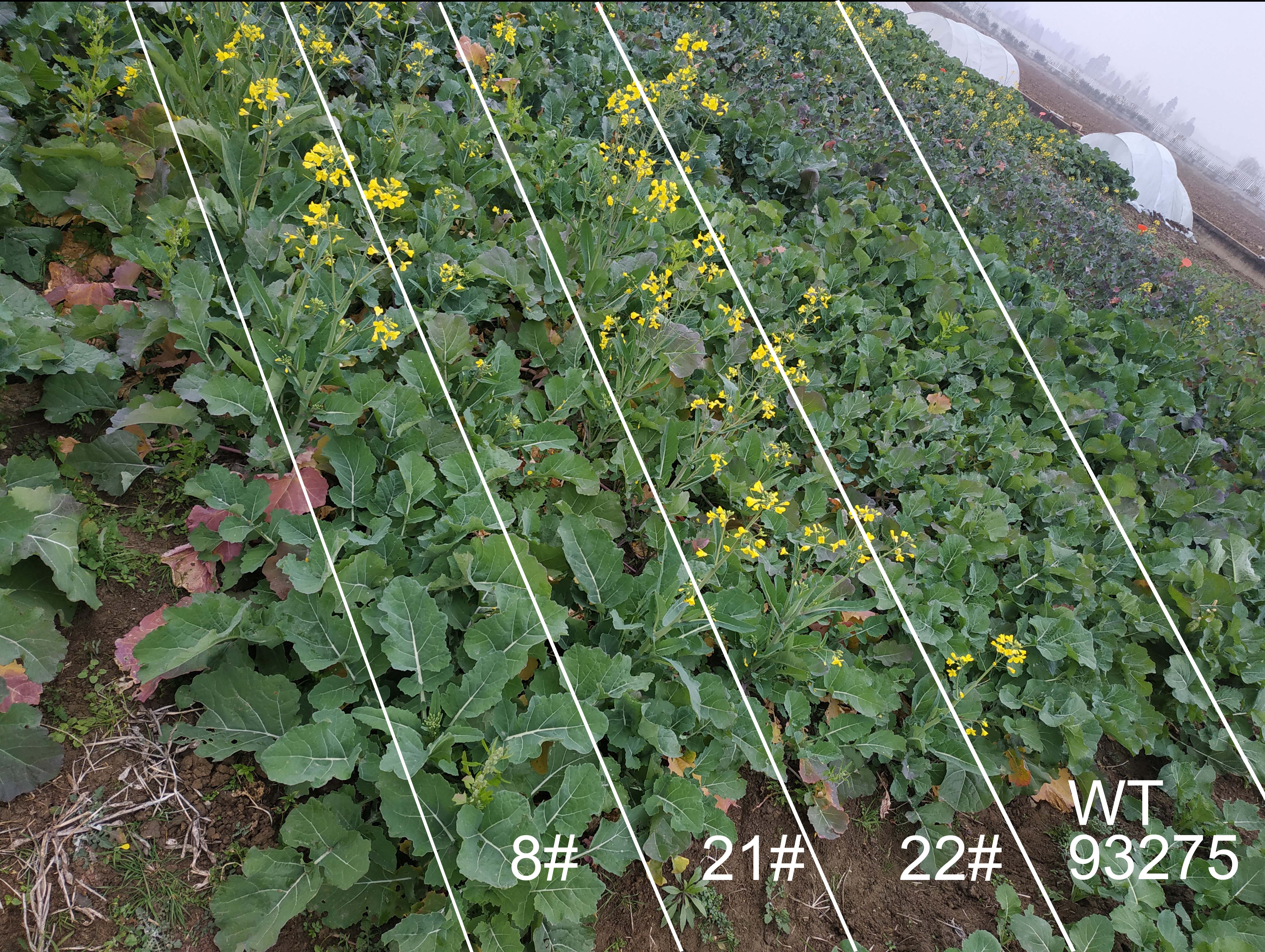

Supplement: Supplementary file 1 [file plants-11-02312-s001.zip › Figure S1.jpg]
